# Expansion of endogenous T cells in CSF of pediatric CNS tumor patients undergoing locoregional delivery of IL13R〿2-targeting CAR T cells: an interim analysis

**DOI:** 10.21203/rs.3.rs-3454977/v1

**Published:** 2023-10-23

**Authors:** Leo Wang, Angela Taravella Oill, M. Blanchard, Melody Wu, Jonathan Hibbard, Sean Sepulveda, Lance Peter, Julie Kilpatrick, Margarita Munoz, Tracey Stiller, Noah Shulkin, Jamie Wagner, Ally Dolatabadi, Monica Nisis, Jennifer Shepphird, Gabriela Sanchez, Chetan Lingaraju, Mishika Manchanda, Heini Natri, Léonce Kouakanou, Grace Sun, Cheryl Oliver-Cervantes, Joseph Georges, Maryam Aftabizadeh, Stephen Forman, Saul Priceman, Julie Ressler, Leonidas Arvanitis, Jennifer Cotter, Massimo D’Apuzzo, Benita Tamrazi, Behnam Badie, Tom Davidson, Nicholas Banovich, Christine Brown

**Affiliations:** City of Hope; Translational Genomics Research Institute; City Of Hope National Medical Center; City of Hope National Medical Center; Beckman Research Institute, City of Hope National Medical Center; City of Hope National Medical Center; Translational Genomics Research Institute; City of Hope National Medical Center; City of Hope National Medical Center; City of Hope; City of Hope National Medical Center; City of Hope; City of Hope National Medical Center; City of Hope National Medical Center; City of Hope National Medical Center; City of Hope National Medical Center; City of Hope National Medical Center; City of Hope National Medical Center; The Translational Genomics Research Institute; City of Hope National Medical Center; City of Hope National Medical Center; City of Hope National Medical Center; City of Hope National Medical Center; City of Hope National Medical Center; City Of Hope National Medical Center; City of Hope; City of Hope National Medical Center; 7Department of Pathology, City of Hope, Duarte, CA, USA.; Children’s Hospital of Los Angeles; City of Hope Comprehensive Cancer Center; University of Southern California; City of Hope; Children’s Hospital Los Angeles; Translational Genomics Research Institute; City Of Hope National Medical Center

## Abstract

Outcomes for pediatric brain tumor patients remain poor, and there is optimism that chimeric antigen receptor (CAR) T cell therapy can improve prognosis. Here, we present interim results from the first six pediatric patients treated on an ongoing phase I clinical trial (NCT04510051) of IL13BBζ-CAR T cells delivered weekly into the lateral cerebral ventricles, identifying clonal expansion of endogenous CAR-negative CD8^+^ T cells in the cerebrospinal fluid (CSF) over time. Additionally, of the five patients evaluable for disease response, three experienced transient radiographic and/or clinical benefit not meeting protocol criteria for response. The first three patients received CAR T cells alone; later patients received lymphodepletion before the first infusion. There were no dose limiting toxicities (DLTs). Aside from expected cytopenias in patients receiving lymphodepletion, serious adverse events possibly attributed to CAR T cell infusion were limited to one episode of headache and one of liver enzyme elevation. One patient withdrew from treatment during the DLT period due to a Grade 3 catheter-related infection and was not evaluable for disease response, although this was not attributed to CAR T cell infusion. Importantly, scRNA- and scTCR-sequence analyses provided insights into CAR T cell interaction with the endogenous immune system. In particular, clonally expanded endogenous CAR^−^ T cells were recovered from the CSF, but not the peripheral blood, of patients who received intraventricular IL13BBζ-CAR T cell therapy. Additionally, although immune infiltrates in CSF and post-therapy tumor did not generally correlate, a fraction of expanded T cell receptors (TCRs) was seen to overlap between CSF and tumor. This has important implications for what samples are collected on these trials and how they are analyzed. These initial findings provide support for continued investigation into locoregionally-delivered IL13BBζ-CAR T cells for children with brain tumors.

Outcomes for patients with recurrent or aggressive brain tumors remain poor, and standard treatments are associated with significant morbidity^1–4^. New therapies are urgently needed, and chimeric antigen receptor (CAR) T cell therapy has shown promise in both pediatric^5–8^ and adult^9–14^ brain tumors. Prior investigations in the field have evaluated the effects of target selection, CAR construction, mode of delivery, and lymphodepletion on CAR efficacy^15–19^. Current pediatric brain tumor CAR T trials target GD2, B7-H3, HER2, and IL13RA2 among other antigens. Some trials incorporate lymphodepletion, and some make use of intraventricular delivery. Much of this work suggests that there is synergy between these factors, but whether particular combinations have generalizable outcomes (e.g., that lymphodepletion affects CAR efficacy equivalently for GD2 and IL13Rα2 targets) remains to be determined, pointing to the importance of disseminating information about these varied clinical experiences without delay. Leveraging inter-trial comparisons to identify general principles of trial design will permit rapid optimization of therapeutic efficacy.

Our previous work has developed CAR T cells targeting the high-affinity IL13 receptor, IL13Rα2, which differs from most other brain tumor-targeted CAR constructs in that its antigen-binding domain is a mutated cytokine rather than a single-chain variable fragment^15,20^. This CAR incorporates an IgG4-based hinge and linker, a CD4-derived transmembrane domain, and a 4–1BB-derived costimulatory domain, and has been extensively investigated in preclinical and adult clinical studies^9,10,14–16^. Our group has shown that CAR T cells are more effective when manufactured from less mature initial T cell populations^21,22^. Additionally, CAR T cells are more effective when administered locoregionally, both in animal models^15,23^ and in patients^10^. Furthermore, preclinical^16,17^ and early clinical evidence indicates that systemic lymphodepletion improves the efficacy of locoregionally-delivered CAR T cells^5,24^. Importantly, IL13BBζ-CAR T cells are capable of mediating a remarkable clinical response in adult glioblastoma patients^10^, and show encouraging evidence of clinical activity in a large cohort of heavily-pretreated adult glioblastoma patients^14^.

Here, we report initial findings from the first six pediatric patients treated with intraventricular IL13BBζ-CAR T cells on a dedicated pediatric clinical trial, testing for the first time the combination of IL13BBζ-CAR T cells with lymphodepletion (NCT04510051). Using single-cell RNA sequence (scRNAseq) and single-cell TCR sequence (scTCRseq) data, we document that CAR T cell therapy leads to infiltration of endogenous T cells into the CSF, and that CAR^−^ CD8^+^ effector T cells, in particular, are clonally expanded in CSF over the course of therapy. These data support the ability of CAR T cells to promote an endogenous cytotoxic T cell response in the CSF; to our knowledge, this has not been described before in human patients. Moreover, these immune dynamics are not observed in the peripheral blood. Additionally, post-therapy tumor analysis in two patients with ependymoma who did not respond to treatment showed that CAR T cells were not recovered from the tumors. Finally, scTCR-seq analysis in one of the two post-therapy tumors revealed that endogenous TCRs expanded in the CSF had higher proportional representation within the tumor than CSF-unexpanded TCRs. This may suggest an improved ability of expanded T cells to access or persist in tumor. Taken together, these early studies highlight the importance of sampling multiple compartments in brain tumor CAR T cell trials, including both CSF and post-treatment tumor, where feasible, so that the prognostic and correlative advantages of these biosamples can be more clearly delineated.

## Results

### Trial Design

The primary objective of this study is to assess the safety and feasibility of repeated intraventricular (ICV) delivery of IL13Rα2-directed CAR T cells after systemic lymphodepleting chemotherapy in children and young adults with IL13Rα2^+^ recurrent or refractory brain tumors (NCT04510051).

Given the demonstrated safety of IL13Rα2-targeting CAR T cells in adults with brain tumors^14^, the documented superiority of locoregional delivery of these cells^15,23^, and the ample evidence indicating that lymphodepletion improves efficacy of locoregionally-delivered solid tumor-targeted CAR T cells^5,24^, our trial design incorporates lymphodepletion before intraventricular IL13BBζ-CAR T cell therapy for pediatric patients with documented IL13Rα2^+^ recurrent or refractory brain tumors.

**Figure 1:**
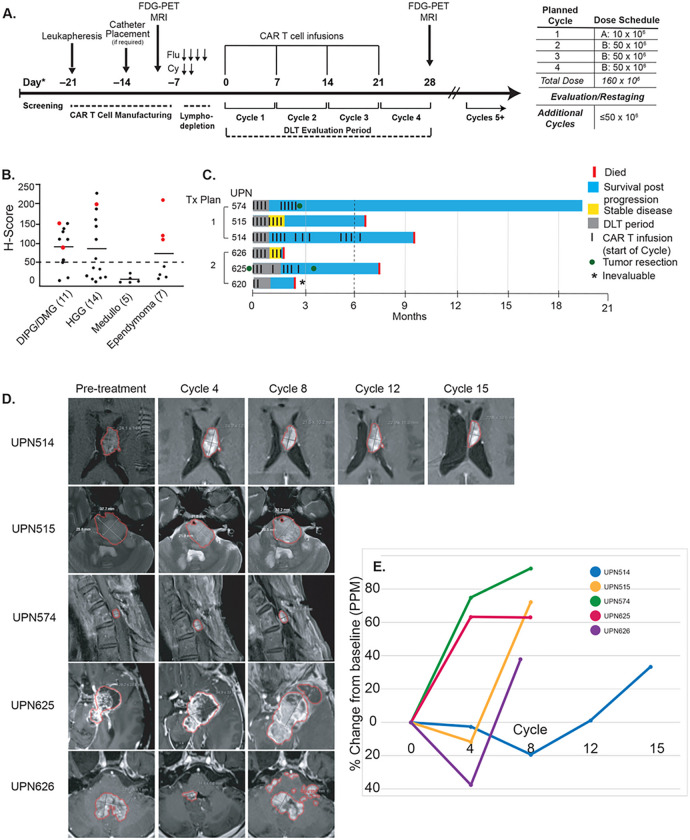
Trial schema and clinical activity of IL13BBζ-CAR T cell therapy. **A)** Patient treatment schema; all 5 patients received the same dose schedule. **B)** Dot plot of screening results (n=37); red indicates treated patients. Trial eligibility requires an H-Score ≥ 50. **C)** Swimmer plots of patient outcomes; 3 patients received CAR T cells alone and 3 received lymphodepletion followed by CAR T cell therapy (UPN620 was inevaluable for disease response). **D)** Longitudinal MRI evaluations showing the largest measured tumor for each patient at the end of the indicated cycle; each cycle begins with a CAR T cell infusion. Malignant tissue is outlined in red for illustrative purposes only. **E)** Line graph showing % change in size, from baseline, of total measurable tumor burden in each patient as calculated by product of perpendicular measurements. This does not align with protocol response, as UPN 514 had unmeasurable progression (leptomeningeal spread).

Patients received weekly intraventricular IL13BBζ-CAR T cell infusions, with the first infusion at a dose of 10 × 10^6^ CAR^+^ T cells and subsequent infusions at 50 × 10^6^ CAR^+^ T cells (Fig. 1A). To verify safety of CAR T cells administered alone, the first three patients on the trial did not receive lymphodepletion. Subsequent patients received four doses of fludarabine (30 mg/m^2^/day) and two doses of cyclophosphamide (500 mg/m^2^/day), ending two days before infusion. After the first four infusions, encompassing the dose limiting toxicity (DLT) evaluation period, patients had the option to continue weekly intraventricular infusions. During active therapy, patients underwent sampling of cerebrospinal fluid (CSF) and peripheral blood (PB) immediately before and 1–2 days after CAR T infusion.

### Patient characteristics and treatment

As of May 16, 2023, 37 patients had been screened for IL13Rα2 by immunohistochemistry (IHC), of whom 18 had an H-Score ≥ 50 for eligibility on the trial (Fig. 1B, **Supplemental Table S1**). Of these, 11 participants completed leukapheresis and product manufacture successfully; there were no failed products (**Figure S1, Table S2**). We report results from the first six patients who received at least one infusion of CAR T cells. One participant (UPN620) received two CAR T doses but developed a catheter-related infection and was taken off protocol therapy. The other five patients received between 7 and 15 intraventricular CAR T cell infusions each.

UPN514 was a 20-year-old Caucasian male with anaplastic PFA ependymoma (Grade 3), originally arising in the fourth ventricle. Prior treatment included treatment on COG ACNS0831, GTR2 arm followed by observation as per randomization. Subsequent imaging showed a recurrent fourth ventricular mass with new nodules in the lateral ventricles and on the septum pellucidum. He underwent re-resection, chemotherapy, and whole brain radiation therapy with boost, after which he also developed spinal metastases; these were treated with Cyberknife irradiation. He received 15 CAR T cell infusions in total, without lymphodepletion.

UPN515 was a 15-year-old Caucasian female with H3K27M-mutant diffuse intrinsic pontine glioma (DIPG) extending to both sides of the midline, with some extension into the upper medulla on the left side. Her prior therapies included radiation therapy, two viral vaccine trials, bevacizumab, and further irradiation. She received eight CAR T cell infusions, without lymphodepletion.

UPN574 was a 23-year-old Caucasian female with multifocal anaplastic PFA ependymoma (Grade 3). Her prior therapies included 4 surgeries and 4 courses of radiation therapy, chemotherapy, and checkpoint antibody therapy. She received nine CAR T cell infusions without lymphodepletion.

UPN620 was a 21-year-old African male with multiply recurrent high-grade glioma previously treated with gross total resection, radiation therapy, tumor treating fields, and chemotherapy. His tumor was H3.3G34R mutant. At first recurrence, he was treated on PNOC013 with cemiplimab after re-resection and radiation therapy. He also received bevacizumab and radiation before enrolling on our trial. He received two CAR T infusions after lymphodepletion, before developing CSF bacteremia requiring cessation of therapy and removal of intracranial hardware.

UPN625 was a 15-year-old Caucasian male originally diagnosed with Grade II ependymoma that underwent aggressive progression. He had a prolonged treatment course over many years incorporating multiple surgeries, several courses of radiation therapy, conventional and metronomic chemotherapy, checkpoint inhibitor therapy, and multiple clinical trials including CCG99703, PBTC-042, AVDL1615, and PNOC023. He received eight CAR T cell infusions after lymphodepletion.

UPN626 was a 15-year-old Latino male with metastatic H3K27M-mutant diffuse midline glioma. Prior treatment included radiation therapy and PNOC023 treatment with ONC-206 followed by palliative radiation to his spinal lesions. He received seven CAR T cell infusions after lymphodepletion.

### Safety

Overall, CAR T therapy with or without lymphodepletion was well-tolerated in these patients, with no DLTs observed during the 28-day observation period encompassing the first four infusions. Some patients experienced limited transient Grade 3 adverse events (AEs), but there were no Grade 4 AEs other than expected cytopenias in patients receiving lymphodepletion. One patient (UPN620) did not complete therapy due to a Grade 3 catheter-related infection not attributable to CAR T cells. The most common toxicities attributed to CAR T at the level of possible or probable were headache and hypertension (Table 1). These toxicities were mild and self-limited, with headache developing metronomically 24–28 hours after each infusion (median 1 day, range 1–2 days) before resolving with minimal intervention. More severe toxicities attributed to CAR T as possible or above included one Grade 3 headache and one Grade 3 increased alanine aminotransferase. Because IL13Rα2 is also expressed on testis^25^, we amended our protocol in September 2022 to collect testosterone data on patients. All subsequent patients have been male so far; these patients were noted to have low pre-treatment testosterone levels, and these levels fluctuated throughout therapy (**Table S3**). In these few patients, an association between CAR T therapy and testosterone level was not observed. Overall, CAR T therapy with or without lymphodepletion seems safe and well-tolerated, supporting the incorporation of lymphodepletion in combination with CAR T cell therapy for malignant brain tumors.

### Treatment outcomes

Among the five patients in this report who completed the DLT evaluation period, three experienced some clinical or radiographic benefit. Two (UPN515 and UPN626) achieved stable disease, and a third (UPN514) experienced a decrease in the size of his largest radiographic lesion concurrent with progression of multifocal and leptomeningeal lesions. Improvements in all cases were transient. In the initial cohort, receiving CAR T cells alone, one in three evaluable participants achieved stable disease (Fig. 1C–E **and Table S4**); the other two had progressive disease. Two (UPN514 and UPN515) had transient tumor shrinkage after 8 and 4 cycles, followed by tumor progression by cycle 12 and 8, respectively (Fig. 1CD). Of the participants who received lymphodepletion prior to CAR T therapy, one in two evaluable patients achieved stable disease (Fig. 1C–E, **and Table S4**). Overall, two of five evaluable patients achieved stable disease and three of five evaluable patients had some tumor shrinkage on MRI. UPN514 did not meet criteria for stable disease, as he had new foci of disease and progression of leptomeningeal disease despite shrinkage of his largest measurable tumor. These preliminary results suggest that IL13BBζ-CAR T cells are capable of providing modest benefit to a significant proportion of pediatric patients with IL13Rα2-expressing brain tumors.

### Correlative studies

#### Peripheral blood and CSF immune landscapes differ over the course of therapy

In nonlymphodepleted patients (UPNs 514, 515, and 574), sufficient mononuclear cells were recovered from the CSF at post-infusion timepoints (d1–2) to permit paired scRNAseq and scTCRseq analysis. Thus, to understand the immune landscape among pediatric patients who received CAR T therapy, we generated scRNAseq data from CSF mononuclear cells for the three nonlymphodepleted patients, and from peripheral blood mononuclear cells (PBMCs) for all five evaluable patients at various cycles of therapy, sequencing at least two cycle timepoints for each patient (**Table S5**). We chose timepoints that coincided with prescheduled MRI studies (Fig. 1C), acknowledging that imaging correlates imperfectly with the kinetics of response, but selecting timepoints where radiographic changes were most apparent to define windows of potential therapeutic response or non-response (Fig. 1D). Radiographic categories corresponding to selected cycles are listed in **Table S6**.

**Figure 2:**
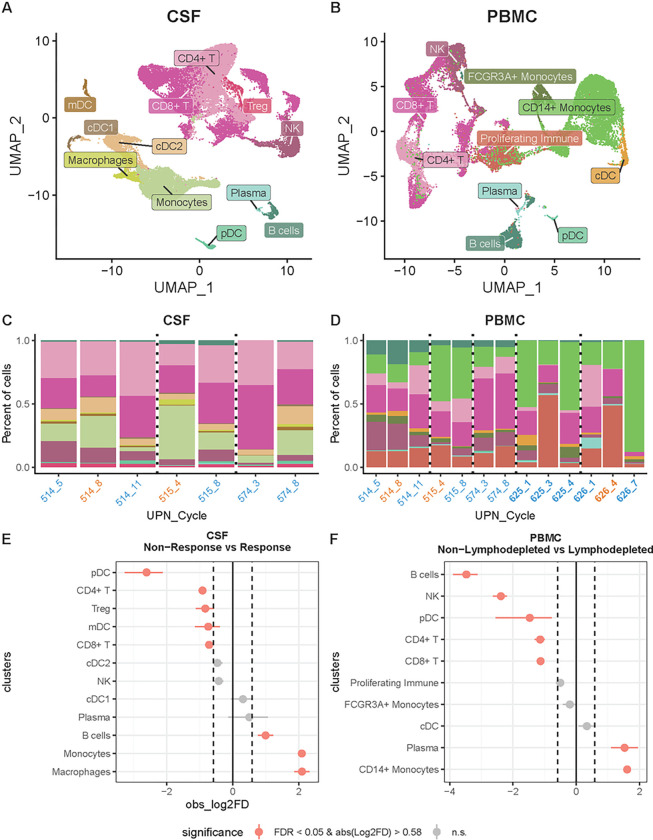
Single-cell landscape of CSF and PBMC in pediatric patients that received CAR T cell therapy. **A)** UMAP of 38,133 cells from CSF across three patients and **B)** UMAP of 24,614 cells from PBMCs across five patients throughout the various cycles of CAR T cell therapy. **C-D)** Stacked bar plot of the proportion of cell types from CSF and PBMCs, respectively, colored according the UMAPs in A and B. Bars are labeled UPN_#: # is the treatment cycle; blue indicates cycles that roughly correspond to nonresponse periods; orange indicates response periods; bold indicates lymphodepletion. **E-F)** Point range plots comparing the proportion of cell types **(E)** during non-response windows and response windows for CSF and **(F)** between patients that received lymphodepletion and patients that did not in PBMC. Colors in A-D represent cell types. Red points in E and F represent cell types with a significant proportional difference between categories and the red horizontal lines represent 95% CIs. Dashed vertical lines represent absolute log2 fold difference (abs(Log2FD)) of 0.58. Significant proportional differences between categories were ones with both FDR <0.05 and abs(Log2FD) of 0.58. Solid vertical line represents abs(Log2FD) of 0. abs(Log2FD) greater than 0 represents cell types at a higher proportion during response windows compared to non-response windows or in patients that received lymphodepletion compared to those who did not.

For the scRNAseq analysis, we recovered in total 38,133 cells across 12 immune cell types in CSF and 24,614 cells across 10 immune cell types in PBMCs (Fig. 2A–B; **Figure S2, S3**). T cells, NK cells, B cells, monocytes, plasma cells, and various types of dendritic cells were identified in both sample types (Fig. 2A–B). Cell types were assigned according to expression of marker genes and top markers for each cluster (**Table S7; Figure S2, S3**), acknowledging that cell phenotypes are context dependent and not always clearly defined by marker genes. We also performed flow cytometry on samples obtained at each cycle timepoint, obtaining flow cytometric data at more timepoints than we had sequencing data for. At timepoints where we had both sequencing and flow cytometry data, the flow data generally correlated with the sequencing data in terms of immune cell proportions (**Figure S4**), However, as the sequencing data is much richer than the flow cytometry data, we focused our downstream analyses on these data.

Analysis of the scRNAseq data demonstrated that although cell types were similar, they were not identical between CSF and PBMCs. For instance, we were able to differentiate CD14^+^ and FCGR3A^+^ monocytes in PBMCs but were unable to differentiate these monocyte subtypes in CSF. Additionally, we identified dendritic cell subtypes (cDC1, cDC2, mDCs), macrophages, and Tregs in CSF but not in PBMCs. In PBMCs, we identified a general proliferating immune cluster that was not found in CSF, which correlated with hematopoietic recovery from lymphodepletion. Among comparable cell types between PBMCs and CSF, we found a higher proportion in PBMCs of NK, plasma, and B cells and a lower proportion of T cells, pDCs, and cDCs (FDR < 0.05 and abs(Log2FD) > 0.58; **Figure S5; Table S8**). Finally, we assessed the degree to which expression programs were coordinated across PBMCs and CSF. Specifically, in these data we observe a distribution of expression levels for any given gene across individuals – both within the CSF and PBMCs. Analyzing each cell type independently, we tested for associations between gene expression levels in CSF and PBMCs, e.g., if higher expression of a given gene in peripheral blood T cells corresponded with higher expression of that gene in CSF T cells. We observed no significant relationship between these two compartments, suggesting gene expression in the PBMCs is not a good proxy for gene expression in the CSF within cell types.

When stratified by patient and cycle, we observed that cell type proportions fluctuate over the course of therapy, although no specific patterns emerged over successive cycles of therapy (Fig. 2C–D). We stratified patients by periods of response and non-response to the extent possible given the constraints of limited imaging timepoints, and found a higher proportion of monocytes, macrophages, and B cells during periods of response compared with periods of non-response in the CSF (FDR < 0.05 and abs(Log2FD) > 0.58; Fig. 2E; **Table S9**). In PBMCs, there were no observed patterns that correlated with response or nonresponse periods. Where there were PBMC samples with and without lymphodepletion, we observed a higher proportion of plasma cells and monocytes among patients who received lymphodepletion (FDR < 0.05 and abs(Log2FD) > 0.58; Fig. 2F; **Table S10**), and a lower proportion of T cells, B cells, NK cells, and dendritic cells (FDR < 0.05 and abs(Log2FD) > 1.1; Fig. 2F; **Table S10**). Overall, through scRNAseq we identified immune infiltrates in the CSF after CAR T cell infusion and cell type proportional differences between response windows and non-response windows, and between patients who did or did not receive lymphodepletion. This analysis revealed post-infusion immune populations in the CSF whose composition changed over time during therapy, and which were distinct from contemporaneous immune populations in the PBMC. Taken together, these data indicate that peripheral blood analyses are unlikely to correlate well with CSF analyses in the context of intraventricular CAR T cell delivery.

### T cell populations and transcriptional profiles differ between CSF, peripheral blood, and cellular product

**Figure 3:**
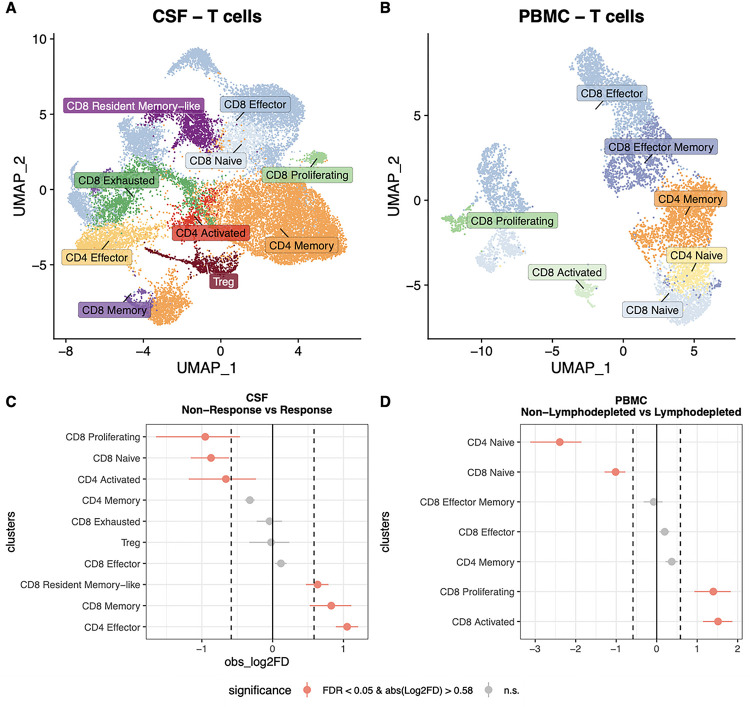
Distinct T cell landscapes in CSF vs. PBMC of patients who received CAR T cell therapy. **A)** UMAP of 24,021 T cells in CSF and **B)** UMAP of 8,052 T cells in PBMCs colored by cell type/state. CSF features several T cell populations that are not prominent in PBMCs. **C-D)** Point range plots comparing the proportion of T cell states **C)** during non-response windows and response windows for CSF, and **D)** between patients that received lymphodepletion and patients that did not in PBMCs. Red points in C and D represent T cell subsets/states with a significant proportional difference between categories and the red horizontal lines represent 95% CIs. Dashed vertical lines represent absolute log2 fold difference (abs(Log2FD)) of 0.58. Significant proportional differences between categories were ones with both FDR <0.05 and abs(Log2FD) of 0.58. Solid vertical line represents abs(Log2FD) of 0. abs(Log2FD) greater than 0 represents cell types at a higher proportion in response windows compared to non-response windows or in patients that received lymphodepletion compared to those who did not.

To understand the T cell response during therapy, we characterized by scRNAseq the T cell landscape and identified 10, 7, and 9 T cell types/states in CSF, PBMCs, and engineered products, respectively (Fig. 3A–B; **Figures S5-S9**). In CSF, we identified CD8^+^ and CD4^+^ T cells, and Treg subsets; in PBMCs and product, we identified CD8^+^ and CD4^+^ T cells, but no Tregs (Fig. 3A–B; **Figure S9A**). CD8^+^ exhausted T cell clusters were identified in both CSF and products but not in PBMCs (Fig. 3A–B; **Figure S9A**). Other T cell states observed included naïve, effector, effector memory, memory, central memory, resident memory-like, activated, and proliferating (Figs. 3A–B; **Figure S9A**).

We found a higher proportion of CD4^+^ effector, CD8^+^ memory, and CD8^+^ resident memory-like states during response windows compared to non-response windows in the CSF (FDR < 0.05 and abs(Log2FD) > 0.58; Fig. 3C; **Tables S9, S10**). Intriguingly, we also found higher proportions of CD8^+^ central memory cells in the engineered products of patients ever experiencing a radiographic response period as compared to non-responding patients (**Figure S9B**). Within the reduced T cell population in the PB of lymphodepleted patients, we observed higher proportions of proliferating and activated CD8^+^ T cells relative to nonlymphodepleted patients (FDR < 0.05 and abs(Log2FD) > 1.3; Fig. 3D; **Tables S11, S12**).

#### Single-cell analysis reveals decreases in CAR^+^ T cell frequency and clonal expansion of CAR^−^ T cells in CSF but not peripheral blood

**Figure 4:**
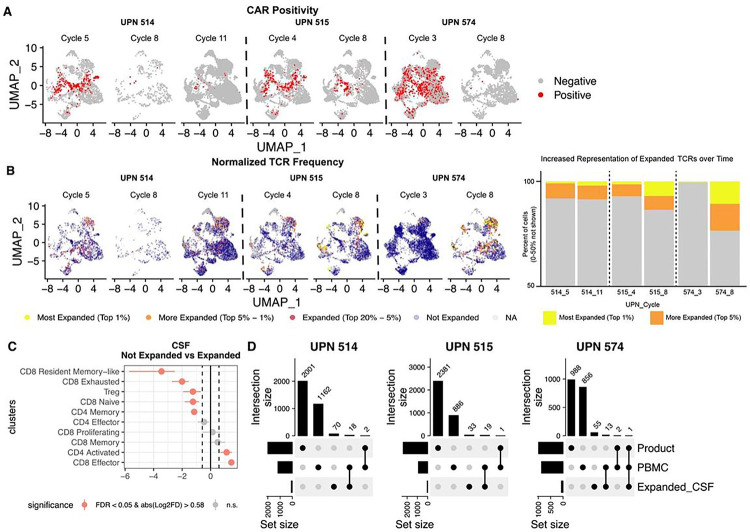
CAR T cell proportion decreases while endogenous T cell clones increase in frequency in CSF over time. **A-B)** UMAP of CSF split by patient (UPN) and cycle. **A)** CAR^+^ T cells (red dots) are located in exhausted, activated, and proliferating clusters and grow less frequent over time in all patients. **B)** Expanded CAR^−^ T cells proportionally increase in all patients over time. Cells are color-coded by normalized TCR frequency category; yellow denotes most expanded (top 1% of all detected TCRs) and orange denotes more expanded (top 1–5% of all detected TCRs) at each timepoint. Expanded T cells cluster largely in activated CD8^+^ effector clusters. Bar graph demonstrates that representation of expanded TCRs increases over time for all patients. **C)** Point range plot comparing the proportion of T cell states in CSF between cells with expanded (Top 20%) and unexpanded TCRs. **D)** Upset plots comparing expanded TCRs in CSF to all TCRs in the product and PBMC for each patient. Red points in C represent T cell subsets/states with a significant proportional difference between categories; red lines represent 95% CI. Dashed vertical lines represent absolute log2 fold difference (abs(Log2FD)) of 0.58. Significant proportional differences between categories were ones with both FDR <0.05 and abs(Log2FD) of 0.58. Solid vertical line represents abs(Log2FD) of 0. abs(Log2FD) greater than 0 represents cell types at a higher proportion among cells with an expanded TCR compared to ones that did not have an expanded TCR.

Using a modification of the standard scRNAseq workflow, we selectively enriched RNA fragments mapping to the CAR construct, enabling us to categorize T cells as CAR^+^ or CAR^−^ (See Methods). Both CAR^+^ and CAR^−^ T cells were detected in CSF and PBMC, with the majority being CAR^−^. The proportion of CAR^+^ T cells was generally greater in CSF than in PBMC across all patients and all timepoints (**Tables S13, S14**). Interestingly, we observed that CAR^−^ T cell proportions increase over time in the CSF (Fig. 4A; **Table S13**), a pattern not observed in PBMC (**Table S14**). CAR^−^ T cells had a higher proportion of CD8^+^ memory, CD8^+^ resident memory-like, and CD4^+^ effector phenotypes, while CAR^+^ T cells had a higher proportion of exhausted and proliferating phenotypes in CSF (FDR < 0.05 and abs(Log2FD) > 0.58; **Figure S10; Table S15**).

To understand the clonal dynamics of T cells during the course of CAR T therapy we generated scTCRseq data for these patients. These data revealed that most expanded TCRs in the CSF are CAR-negative (Fig. 4A–B; **Figure S11**). Across cycle timepoints there was an increase in proportion of the most expanded TCR clonotypes in all patients; this was true for all patients when comparing early and late timepoints although the magnitude of increase was variable (Fig. 4B). Among cells with expanded TCRs, we observed a higher proportion of CD8^+^ effector and CD4^+^ activated cells compared to cells that were not expanded (FDR < 0.05 and abs(Log2FD) > 0.58; Fig. 4C; **Table S16**). Interestingly, with the exception of one TCR clonotype in one patient (UPN 574), expanded TCRs in the CSF were not observed in the product, and were infrequently observed in PBMCs (Fig. 4D). Overall, our characterization of the T cell state landscape showed that endogenous CAR^−^ T cell clones were increasingly prevalent in the CSF over the course of CAR T cell therapy, and that these expanded T cells are mostly activated and effector CD8^+^ T cells. This suggests that CAR T cell therapy may entrain and/or expand endogenous activated effector T cells in the CSF.

### Post-treatment tumor resection

**Figure 5:**
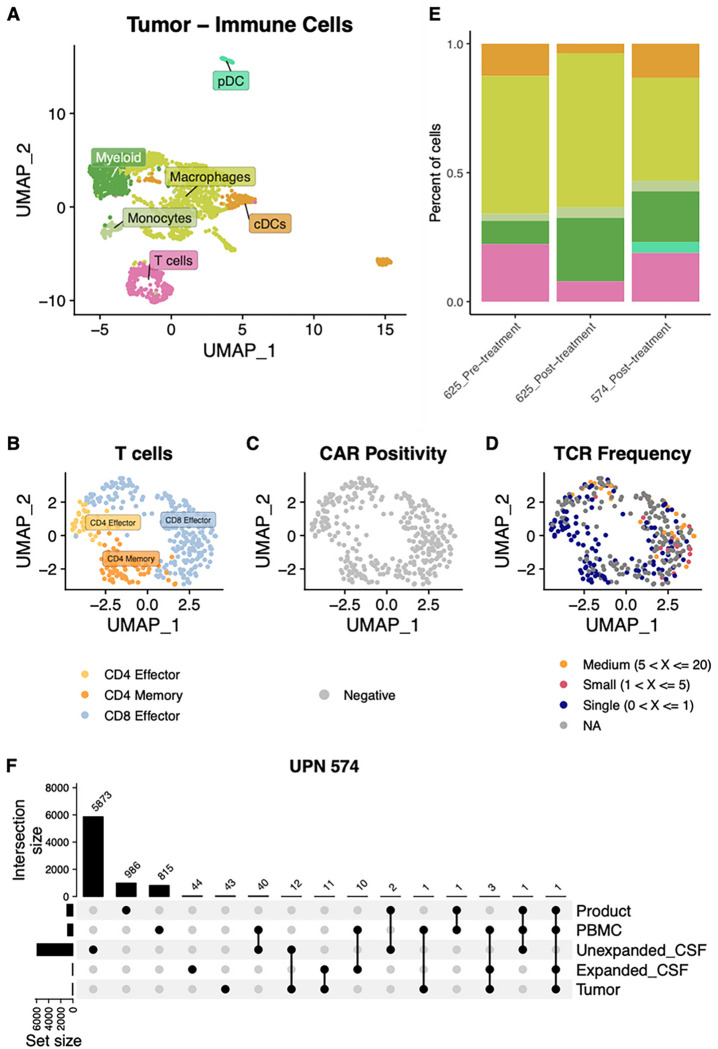
Characterization of immune cells in tumor. UMAP of **A)** 2,085 immune cells from tumor across two patients (UPNs 574 and 625). UMAPs of 313 recovered T cells colored by **B)** T cell state, **C)** CAR positivity, and **D)** TCR frequency. TCR data were only recovered from 625 pre- and 574 post-treatment tumor so all cells for 625 post-treatment tumors are colored gray. **E)** Stacked bar plot of cell type proportions for each sequenced tumor sample. **F)** Upset plot comparing all TCR clonotypes among tumor, CSF, PBMC, and product for UPN 574. CSF TCRs are separated by whether they have an expanded or unexpanded TCR frequency. 11 TCRs found in the tumor overlapped with expanded TCRs found in the CSF (of 69 total expanded TCRs in CSF); 12 TCRs found in the tumor overlapped with unexpanded TCRs found in the CSF (of 6,128 total unexpanded TCRs in CSF).

Two patients, UPN574 and UPN625, underwent tumor resection after CAR T therapy. UPN574 had a resection one week after Cycle 9 of CAR T therapy, and UPN 625 underwent resection one month after Cycle 8 of CAR T therapy. Both patients had PFA ependymoma, and neither experienced a radiographic response during treatment. UPN574 did not undergo lymphodepletion, whereas UPN625 did. Both of their resected tumors had low levels of T cell infiltration and high levels of myeloid infiltration post-CAR therapy by immunohistochemistry, similar to pre-therapy, as well as preserved expression of IL13Rα2. These features have been well described as a poor prognostic indicator associated with recurrence and with subgroup A ependymoma^26–28^. Interestingly, UPN574 also demonstrated metronomic spikes in inflammatory cytokines in the CSF, indistinguishable from patients who experienced a response period (**Figure S12**). Unfortunately, CSF samples from UPN625 were insufficient for single-cell or cytokine analyses. For UPN574, however, clonal expansion of CAR^−^ CD8^+^ T cells in the CSF was a prominent feature and indistinguishable from the kinetics observed in the CSF of UPN514 and UPN515 (Fig. 4). Resected tumors were submitted for single-cell sequencing analysis (**Figure S13**), and we recovered a total of 2,085 immune cells across 5 cell types and one general myeloid population in tumors from those two patients (Fig. 5A). Of the 313 intratumoral T cells observed, we observed both CD4^+^ and CD8^+^ T cells, but all observed T cells were CAR^−^ (Fig. 5B and 5C). Some TCRs were observed to be expanded, or to overlap with TCRs observed to be expanded in CSF (Fig. 5D). For UPN625, where we obtained scRNAseq for pre- and post-treatment tumors, we observed lower proportions of cDCs and T cells and higher proportions of myeloid cells in post-treatment tumor compared to pretreatment tumor (Fig. 5E). For UPN574, we obtained scTCRseq for post treatment tumor. This analysis revealed 11 TCRs in tumor that were expanded in CSF, and 12 TCRs in tumor that were not expanded in CSF (Fig. 5F). Though the number of tumor TCRs was roughly equal in each population, the proportion of TCRs in tumor that overlap with expanded TCRs in CSF is substantially higher (11 out of 69) than the proportion of TCRs in tumor that overlap with unexpanded TCRs in CSF (12 out of 6128; Fig. 5F). TCRs that overlapped with expanded clones in CSF retained the activated effector transcriptional signature seen in those cells in CSF. Taken together, these data suggest that endogenous CAR^−^ T cells can traffic between CSF and tumor, and that expanded endogenous T cells may do this more effectively than unexpanded T cells. CAR^+^ T cells were not detected in post-therapy tumors, which may be due to poor access to the tumor, poor persistence in the CNS, or both. Notably, CAR^+^ T cells were also not prominently detected in the CSF sample from UPN574 obtained closest to resection (Cycle 8, **Table S13**). These findings highlight the importance of evaluating both the CSF and the tumor itself, as some features of CSF may correlate with features in the intratumoral microenvironment whereas others may not. Additionally, these data underscore the importance of local tumoral obstacles in limiting the potential efficacy of adoptive cellular therapies for solid tumors.

## Discussion

We present results from the first six pediatric patients treated with IL13BBζ-CAR T cells, showing the therapy to be safe and well-tolerated. Three patients experienced modest clinical or radiographic benefit. Although analysis of PBMCs was not informative, single-cell RNA analysis of CSF mononuclear cells post CAR T infusion identified immune infiltrates that evolved over the course of therapy. B cell and macrophage proportions appeared to increase during response window periods; these changes must be confirmed in future patients. Strikingly, all patients analyzed experienced increases in the proportion of T cells in CSF that were CAR^−^ over time, with the proportion of CAR^+^ T cells decreasing over time. The CSF-infiltrating CAR^−^ T cells clustered into CD8^+^ memory, CD8^+^ resident memory-like, and CD4^+^ effector populations (**Figure S10**); in contrast, CAR^+^ T cells clustered into exhausted and proliferating phenotypes. Intriguingly, T cell receptor (TCR) sequencing identified clonal expansion of CD8^+^ CAR^−^ T cells, but not CAR^+^ T cells, in all evaluated patients over time. Unfortunately, these analyses were not feasible in patients who received lymphodepletion, for technical reasons. In patients not receiving lymphodepletion, TCRs from expanded clones were generally not identified in the infusion product. Taken together, these findings suggest that CAR T cell therapy entrains endogenous CD8^+^ T cells to infiltrate the CSF, and that a subset of these T cells undergoes clonal expansion over the course of therapy.

T cell clonal diversity in peripheral blood and tumor have been extensively studied (reviewed in ^29^ and elsewhere), but to our knowledge this is the first report of clonal expansion in the CSF of human patients receiving immunotherapy. The implications of TCR diversity and clonality in the context of immunotherapy are clearly dependent on tumor type, therapy, and other factors, so it is difficult to extrapolate what clonal expansion in this trial might indicate. Recently, research has suggested that TCR convergence, representing a response to particular antigens, may be important^30,31^; studies are ongoing to determine the antigen specificity of expanded TCRs identified in our patients. Additionally, we continue to study this phenomenon in patients receiving lymphodepletion. Taken together, these findings may represent a first step in understanding how to use adoptive cellular therapies to promote endogenous antitumor immune responses.

Consistent with other studies, including our extensive experience with adult glioblastoma patients receiving IL13BBζ-CAR T cells^10,14^, and consistent with patients’ metronomic clinical symptoms, intraventricular delivery of CAR T cells was associated with post-infusion increases in CSF inflammatory cytokines such as IFNγ, IL-6, IL-8, CCL4, and CXCL10 (**Figure S12**). These spikes were detected the day after each infusion and dissipated before the next CAR T infusion. Moreover, studies with IL13BBζ-CAR T cells in adult glioblastoma finds that levels of interferon pathway cytokines IFNγ, CXCL9, and CXCL10 are positively associated with clinical response^14^. Peripheral blood serum cytokine levels, in contrast, were much more stable both within and between cycles; this is also similar to what has been reported by other groups ^5,7,14,15^.

Our analysis of residual tumors after CAR T therapy in two patients (UPN 574 and 625), is preliminary and should not be overinterpreted. However, our data suggest that endogenous immune cells, particularly clonally expanded CD8^+^ effector cells found in the CSF, may preferentially gain access to the tumor as compared to unexpanded CD8^+^ cells. CAR^+^ T cells were not recovered from post-therapy tumor, suggesting that they may have poor access to or persistence in the tumor microenvironment in nonresponding patients.

Taken together, these preliminary results underscore the importance of understanding the strengths and limitations of clinical trial correlative analyses in various compartments. We find that peripheral blood is not a good proxy for CSF in the context of intraventricular CAR T cell delivery; the extent to which CSF accurately reflects tumor is also unclear. In our analysis, clonally expanded T cells in the CSF were recovered from the tumor; however, unexpanded CSF T cells were very infrequently recovered from tumor. CAR^+^ T cells were not recovered from tumor at all, reinforcing the contention that significant barriers exist between the CSF and the tumor microenvironment.

This preliminary report of the first pediatric patients treated with IL13Rα2-targeted CAR T cells at a single institution indicates that intraventricular CAR T cell therapy is well-tolerated in children with recurrent or refractory brain tumors, both with and without preceding lymphodepletion. Additionally, this is, to our knowledge, the first report showing that intraventricular delivery of CAR T cells is associated with infiltration and clonal expansion of endogenous T cells in the CSF. Further studies are ongoing to determine how lymphodepletion affects this infiltration, as well as whether the expanded T cells are tumor specific. These data have clear implications for further avenues of study in ongoing trials, and could only be generated by coupling longitudinal CSF, peripheral blood, and tumor sampling with single-cell techniques. Importantly, deep interrogation of post-therapy tissue will likely be critical for improving cellular therapies; every effort should be made to partner with patients and families to gain this precious knowledge. Dense sampling of multiple compartments, as well as single-cell TCR sequencing and single-cell analysis capable of discriminating between CAR-positive and CAR-negative T cells, should be implemented across immunotherapy clinical trials.

## Methods

### Oversight and informed consent

The clinical protocol and all amendments were approved by the City of Hope Institutional Review Board. Ongoing review is performed by a data and safety monitoring committee (DSMC) as well as a cancer protocol review and monitoring committee (CPRMC). Patients and/or guardians provided written informed consent before tissue screening and leukapheresis, and a separate written informed consent before enrollment on the therapeutic portion of the trial. Minor assent was obtained when appropriate.

### Clinical trial design

This phase I study assesses the safety and feasibility of repeated intraventricular (ICV) delivery of IL13Rα2-directed CAR T cells after systemic lymphodepleting chemotherapy in children and young adults with IL13Rα2^+^ recurrent or refractory brain tumors (NCT04510051). Patients receive at least 4 weekly IL13Rα2-CAR T cell infusions, with the first infusion at a dose of 1 × 10^7^ CAR^+^ T cells and subsequent infusions at 5 × 10^7^ CAR^+^ T cells (Fig. 1). To verify that CAR T cells administered alone were safe and well-tolerated, the first three patients on the trial did not receive lymphodepletion. Subsequent patients receive intravenous cyclophosphamide (500mg/m^2^ per day × 2 days, on days - 5 and - 4) and fludarabine (30mg/m^2^ per day × 4 days, on days - 5 through - 2). The first 3 participants on treatment plan 1, and the first 3 participants of each disease type on treatment plan 2 were staggered through 28 days. Secondary objectives include evaluating persistence and expansion of CAR T cells and endogenous immune cells in peripheral blood (PB) and cerebrospinal fluid (CSF), evaluating cytokine levels in PB and CSF, and evaluating potential efficacy and biologic response to treatment.

Children and young adults under 21 years of age at time of initial diagnosis (ages 4–25 at the time of enrollment) with histologically-confirmed malignant brain neoplasms are eligible, with radiographic evidence of recurrence or progression after the end of the initial conventional therapy, including radiation. Additional requirements include IL13Rα2 tumor expression by immunohistochemistry (IHC; H-score > 50) and a Karnofsky or Lansky performance score ≥ 60, except for loss of mobility due to disease.

The trial has been approved by the institutional review board at City of Hope and is conducted under a Food and Drug Administration (FDA) Investigational New Drug application in accordance with International Council for Harmonization Good Clinical Practice guidelines. The first patient was treated on the trial December 14, 2020. Further details of the trial design are provided in the Supplementary Appendix.

### IL13Ra2-targeted CAR design and manufacture

The design of our CAR construct has previously been described ^9,15,16^. Briefly, the construct comprises a modified cytokine-based antigen binding domain coupled to an IgG4Fc-based hinge and linker region, followed by a CD4 transmembrane domain, 4–1BB costimulatory domain, and CD3z signaling domain. This construct was lentivirally transduced into a naïve/memory T cell pool, as previously documented ^15,16^. For the six patients described here, product manufacturing characteristics included are detailed in **Table S2**.

### scRNA and scTCRseq library preparation and sequencing

Single-cell library preparation was carried out on 33 clinical patient derived samples according to 10X Genomics Chromium Next GEM Single Cell 5’ Reagent Kits v2 (Dual Index) CG000331 Rev A. 13 Peripheral blood mononuclear cells (PBMC), 5 CAR-T products, 5 tumors, and 10 CSF samples were placed into 6 batches yielding GEX, TCR V(D)J, feature barcode, and CAR transcript enrichment libraries (**Table S5**). Sequencing was achieved on the NovaSeq6000.

Batches 37, 38, 39, and 40 used Biolegend Total Seq C Human hashtag antibodies to barcode product and PBMC samples and allow for later patient pooling and deconvolution ^32^. PBMC, CAR-T product, and tumor samples were sorted for viability using Calcien-AM live stain and sorted into a collection tube for further processing. All CSF and tumor samples, and 1 CAR-T product, were loaded directly to individual GEM reactions. CSF samples did not undergo Calcein-AM staining or preprocessing and were loaded to GEM directly following cryopreservation thaw. CAR transcript enrichment libraries were performed on batch 37 with a series of 3 custom nested primers starting at cDNA amplification, then subsequent batches used a series of 4 nested CAR primers included at reverse transcription to further enrich lowly transcribed targets. In addition, batches 37 and 38 had 3 non-hashed CSF samples run in a separate Gel Bean-in-Emulsion (GEM) reaction. Batch 42 included 4 non-hashed CSF samples, 3 duplicate samples from batch 37 and one new patient CSF sample. Duplicate libraries were generated in order to saturate CAR transcript diversity. Batch 39 contained 4 hashed samples and 2 non-hashed tumor samples. Batch 40 contained 3 hashed samples and 1 non-hashed tumor sample and 1 non-hashed product sample. Batch 41 contained no hashing and 2 non-hashed tumor samples. Hashed live single cell patient samples were pooled at 10,000 cells per sample and loaded to GEM. Multiplexed GEM reactions were pooled and loaded at 30–50k cells per GEM to maximize yield and restrict unidentifiable doublet counts using Satija Lab Multiplexing Cost Calculator ^33^. Cell count validation using Illumina Iseq100 provided a recommended sequencing depth for the relevant library type and pooled accordingly to NovaSeq6000.

Gene expression libraries were run with CellRanger v5.0.1 using the count function and default settings ^34^. GRCh38 Ensembl 98 with the CAR construct added was used as the reference. For the samples that were multiplexed (**Table S5**), we used the same approach as above but added the --include-introns flag and provided the antibody panel (Total Seq C).

### scRNA-seq data analysis

Single cell gene expression matrices were processed in R v4.2.0 ^35^ using Seurat v4.1.1^36^. Sample-type specific filtering was performed for the gene expression matrices using the following filtering thresholds 1) CSF: pt_mt = 10, nFeature = 650, nCount = 1200, 2) PBMCs: pt_mt = 10, nFeature = 650, nCount = 1200, 3) product: pt_mt = 10, nFeature = 1300, nCount = 2500, and 4) tumors: pt_mt = 10, nFeature = 1500, nCount = 2300 (**Figure S14**). After filtering, we estimated and corrected the gene expression matrices for ambient RNA using SoupX v1.6.2 ^37^. For the samples that were multiplexed (**Table S5**), we demultiplexed using HTODemux in Seurat and converted the demultiplexed Seurat object to 10x sparse matrices using DropletUtils v1.16.0 write10xCounts ^38^ prior to running SoupX. Contamination fraction was automatically estimated for each sample using the autoEstCont function and gene expression matrices were corrected using the adjustCounts function in SoupX. Cell cycle scores were then assigned using the CellCycleScoring function in Seurat and regressed out during normalization. Normalization was performed using SCTransform v2^39,40^. Mitochondrial and ribosomal genes were removed prior to integration and downstream analyses.

We used the rPCA method for integration followed by PCA dimensionality reduction on the top 2500 most variable genes and visualization using Uniform Manifold Approximation and Projection (UMAP). The number of PCs used in the UMAP visualizations were determined by using an in-house script that selects the PC where change of percent of variation is less than 0.1%. Nearest-neighbor graphs were constructed using the PCA reduction and cells were clustered to various resolutions (0.1, 0.2, 0.3, 0.5, 0.8, and 1) using the Louvain algorithm. For samples that were multiplexed (PBMCs and product), we integrated by sequence batch, while for CSF and Tumor, we integrated by individual sample. CSF, PBMCs, product, tumors, were integrated separately, CSF and PBMC samples were integrated together.

Cell type annotations were performed by visualizing gene expression of known marker genes and top markers for each cluster (**Table S17; Figures S2, S3**). Sub-clustering was performed where needed using FindSubCluster function in Seurat. Top markers were identified using Presto’s v1.0.0 Wilcoxon rank sum test to identify top markers for each cluster ^41^.

T cell states were further annotated for CSF, PBMC, and product. We first subsetted T cell clusters from PBMC and CSF objects and performed integration, dimensionality reduction, clustering, and visualization as described above. Clusters with expression of non-T cell genes (for example, *LYZ*) were removed, data were re-clustered, and the UMAP was re-generated. Similar to the cell type annotations, T cell states were identified by visualizing gene expression of known marker genes and top markers for each cluster (**Table S7; Figures S6-S8**). Additionally, we ran gene set variation analysis on single cell data on RNA using scGSVA v0.0.13 ^42^ to identify clusters with enrichment of gene expression for the different T cell states.

Cell type proportion differences were tested between periods of response and non-response, across cycle time points for each patient, between CAR^+^ and CAR^−^ T cells, and between expanded and unexpanded T cells using scProportionTest v0.0.0.900 ^43^ and visualized using dittoseq v.1.8.1^44^. To compare the differences in proportions of cells between scRNA-seq samples we used scProportionTest, which facilitates permutation tests to obtain p-values for each cluster and bootstrapping to obtain confidence intervals. Response categories were based on radiographic measurements of patient’s tumors at cycles 4, 8, and 11. We grouped samples into either response or non-response categories. Non-response periods categories included stable, pseudoprogression, or progression. If a sequence sample fell between cycles, the response of the later time point was assigned to the sample (**Table S6**). CAR^+^ T cells were identified as an annotated T cell having three or more reads supporting the CAR construct (IL13OP).

To assess whether expression in PBMC is a good proxy for expression in CSF, we tested whether sample level expression in PBMC is associated with sample level expression in CSF (**Figure S5**). Linear models were run in R, where gene expression in CSF samples was the outcome variable and gene expression in PBMC samples was the predictor variable (lm(CSF ~ PBMCs)). For each sample and cell type we pseudobulked by calculating the mean counts for all of the cells for each UPN, cycle, and compartment (CSF or PBMC) combination. Any sample with less than 10 cells for a cell type were not included in the analysis and any cell type with less than three PBMC-CSF matched samples were excluded. We additionally removed genes where at least half of the samples had no expression. FDR correction was performed on each cell type.

### scTCRseq data analysis

scRepertoire v1.7.2 ^45^ was used on the filtered contig annotation files to identify clonotypes. We required both alpha and beta chains to be present and used the strict calling method (VDJC gene and CDR3 nucleotide present) when calling clonotypes. TCR frequency (total number of observed cells with the clonotype) were calculated for each UPN, cycle, and sample type combination. Clonotypic information generated from scRepertoire was added to the single cell Seurat objects using the combineExpression function. TCR frequencies were normalized by the total number of T cells for each UPN, cycle and sample type combination. Expanded TCRs were defined as having a TCR count greater than one and having a normalized TCR frequency in the top 20% of normalized TCR frequencies across all samples. We further categorized TCRs as “expanded” with a normalized TCR frequency between the top 20–5% of the distribution, “more expanded” with a normalized TCR frequency between the top 5–1% of the distribution, and “most expanded” with a normalized TCR frequency at the top 1% of the distribution.

## Figures and Tables

**Table 1:** Safety characteristics

Treatment Plan 1				
Body system	Event	Grade		
		2	3	4
Gastrointestinal disorders	Nausea	1	0	0
General disorders and administration site conditions	Fatigue	1	0	0
	Fever	1	0	0
Nervous system disorders	Headache	3	0	0
	Spasticity	1	0	0
Vascular disorders	Hypertension	1	0	0
**Treatment Plan 2 - Related to CAR T treatment**				
Gastrointestinal disorders	Nausea	1	0	0
	Vomiting	1	0	0
Investigations	Alanine aminotransferase increased	0	1	0
Nervous system disorders	Dysarthria	1	0	0
	Headache	0	1	0
Vascular disorders	Hypertension	2	0	0
**Treatment Plan 2 - Related to lymphodepletion**				
Blood and lymphatic system disorders	Anemia	1	0	0
Gastrointestinal disorders	Dry mouth	1	0	0
	Nausea	1	0	0
	Vomiting	2	0	0
General disorders and administration site conditions	Fatigue	1	0	0
Infections and infestations	Catheter-related infection	0	1	0
Investigations	Lymphocyte count decreased	0	0	3
	Neutrophil count decreased	2	0	1
	White blood cell count decreased	0	2	1

## Data Availability

Scripts for processing sequencing data will be made publicly available on GitHub at https://github.com/Banovich-Lab/19130_Pediatric_CART. Aggregated patient data, once adequately deidentified to protect patient privacy, will be made available upon request. TCRseq and scRNAseq data from patient samples reported here will be deposited in GEO under an accession number which will be included at the time of publication.
